# Enhancing Perovskite Solar Cell Performance through Propylamine Hydroiodide Passivation

**DOI:** 10.3390/nano14171416

**Published:** 2024-08-29

**Authors:** Fulin Sun, Ting Zhu, Chenhui Zhang, Yi Dong, Yuzhu Guo, Dan Li, Fangtian You, Chunjun Liang

**Affiliations:** 1 Key Laboratory of Luminescence and Optical Information, Ministry of Education, Institute of Optoelectronic Technology, Beijing Jiaotong University, Beijing 100044, Chinaftyou@bjtu.edu.cn (F.Y.); 2 Department of Physics, School of Physical Science and Engineering, Beijing Jiaotong University, Beijing 100044, China

**Keywords:** halide perovskite, perovskite solar cells, defect passivation, iodide, hysteresis

## Abstract

In recent years, the power conversion efficiency of perovskite solar cells has increased rapidly. Perovskites can be prepared using simple and cost-effective solution methods. However, the perovskite films obtained are usually polycrystalline and contain numerous defects. Passivation of these defects is crucial for enhancing the performance of solar cells. Here, we report the use of propylamine hydroiodide (PAI) for defect passivation. We found that PAI can result in higher-efficiency cells by reducing the defects and suppressing non-radiative recombination. Consequently, n-i-p perovskite solar cells with a certificated efficiency of 21% were obtained. In addition, PAI exhibited excellent performance in p-i-n devices by serving as a buried interface layer, leading to an improved efficiency of 23%.

## 1. Introduction

As one of the most promising photovoltaic materials, organic–inorganic hybrid perovskite possesses many unique optoelectronic properties, such as tunable bandgaps, high absorption coefficients, and long carrier diffusion lengths [1,2,3,4,5]. By adjusting the composition of perovskite films, controlling perovskite crystallization, and optimizing transport layers, researchers successfully improved the power conversion efficiency (PCE) of perovskite solar cells (PSCs) to a height that is now comparable to that of silicon solar cells [6,7,8]. Currently, the most used method for preparing perovskite is solution-processed, resulting in polycrystalline films with numerous defects, such as grain boundaries and crystalline defects [9,10,11]. These defects can lead to the accumulation of high-density trap states, causing carrier recombination and related losses in open-circuit voltage (V_oc_) [12,13]. Therefore, the passivation of surface and grain boundary defects in perovskite materials is crucial for further improving the performance of perovskite solar cells [14,15,16].

Many effective surface passivation methods have been reported for use in perovskite solar cells. For example, excess lead iodide (PbI_2_) in perovskite films can suppress charge recombination at the film surface and grain boundaries [17,18,19]. Some studies have introduced semiconductor molecules containing Lewis acid or base functional groups to passivate trap states at grain boundaries and suppress over-crystallization [18,20,21,22,23]. Alkyl ammonium halide salts are also popular for the passivation of perovskite films, as they are fundamental materials for synthesizing perovskite [16,24,25,26]. These materials can effectively passivate cation and anion defects on the surface, significantly enhancing device performance. Some linear alkaneamine functionalities, such as ethylammonium (EA^+^), n-butylammonium (BA^+^), guanidinium (Gua^+^), and octylammonium (OA^+^), have been reported to significantly improve device performance by acting as passivation layers in perovskite solar cells [24,27,28,29]. Additionally, alkylammonium halides with benzene rings, especially phenethylammonium iodide (PEAI), exhibit exceptional performance in defect passivation [30,31]. You et al. used PEAI as a passivating material for perovskites and fabricated a device with an efficiency of 23.32% [31]. However, not all passivating materials are effective in both conventional (n-i-p) and inverted (p-i-n) device configurations. 

In this study, we employed an organic halide salt, propylamine hydroiodide (CH_3_CH_2_CH_2_NH_3_I, PAI) for the post-treatment of perovskite to suppress the surface defects of perovskite polycrystalline films for efficient solar cells. The performance of the solar cells significantly improved with the introduction of PAI, especially in terms of open-circuit voltage (V_oc_) and fill factor (FF). As a result, a 21% certified n-i-p PSC was achieved. Moreover, PAI also showed outstanding performance in inverted PSCs, with the efficiency increasing from 21.7% to 23% after PAI optimization.

## 2. Experimental Section

### 2.1. Materials

Lead iodide (PbI_2_, 99.9%), lead bromide (PbBr_2_, 99.9%), formimidamide hydroiodide (FAI, 98%), methylammonium bromide (MABr, 98%), methylamine hydrochloride (MAC), 98%), and cesium iodide (CsI, 98%) were purchased from TCI. SnO_2_ nanoparticle dispersion (15 wt.%) and tris-(8-hydroxyquinoline) aluminum (Alq_3_) were purchased from Sigma-Aldrich. PCBM (99.5%) was purchased from Nano-C. Propylamine hydroiodide (PAI, 99.5%), poly[bis(4-phenyl)(2,4,6-trimethylphenyl)amine] (PTAA), lithium bis(trifluoromethanesulphonyl)imide (Li-TFSI), and 4-tert-Butylpyridine (tBP) were purchased from Xi’an Yuri Solar Co., Ltd. (Xi’an, China). All solvents, dimethyl sulfoxide (DMSO, extra dry), N, N-dimethylformamide (DMF, extra dry), toluene, chlorobenzene (CB), 1,2-dichlorobenzene (DCB), ethanol, and isopropanol (IPA) were purchased from Acros (Shanghai, China).

### 2.2. Device Fabrication

**Figure 1:**
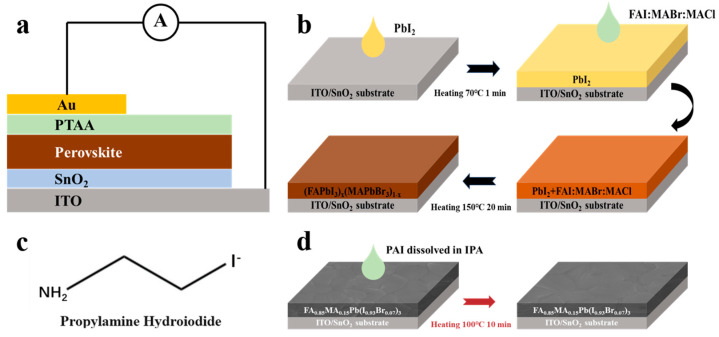
Schematic diagrams of (**a**) the device structure of n-i-p perovskite solar cells; (**b**) the two-step process for perovskite fabrication; (**c**) the chemical structure of the PAI molecule; and (**d**) the procedure of PAI post-treatment.

The configuration of the n-i-p device is presented in Figure 1a. Herein, the SnO_2_ layer was directly deposited on a cleaned bare indium tin oxide (ITO)-coated glass, functioning as an electron transport layer (ETL). SnO_2_ nanoparticle dispersion was diluted with deionized water to 2.67 wt.% and spin-coated on glass/ITO substrates at 4000 rpm for 30 s in ambient air. Then it was annealed at 150 °C for 30 min. After the deposition of SnO_2_, the substrates were moved into a N_2_ glovebox. The perovskite film was prepared using a two-step method, with a composition of FA_0.85_MA_0.15_Pb(I_0.93_Br_0.07_)_3_ according to our former work [32,33]. As shown in Figure 1b, first, 1.3M PbI_2_ in a mixed solvent of DMF and DMSO with a volume ratio of 19:5 was spin-coated on the SnO_2_ layer at 2000 rpm for 30 s, then annealed at 70 °C for 2 min; second, after the PbI_2_ cooled down, the mixture solution (60 mg FAI, 6 mg MABr, and 6 mg MACl in 1 mL IPA) was spin-coated on the PbI_2_ layer at 2000 rpm for 30 s to form a perovskite precursor film. Then, it was taken out of the glovebox and annealed at 150 °C for 20 min in ambient air (30–40% humidity) and converted into a perovskite film. After perovskite growth, the film was transferred back to the glovebox, and PAI was employed for the post-treatment of perovskite films. Figure 1c illustrates the chemical structure of the molecule. As shown in Figure 1d, the PAI solution (dissolved in IPA with different concentrations) was spin-coated on perovskite film at 5000 rpm for 30 s and heated at 100 °C for 10 min in N_2_. The PTAA ($\bar{M_{n}\;}=$ 17,400) solution was coated at 3000 rpm for 30 s, and 1 mL PTAA/toluene (10 mg/mL) solution was employed with the addition of 7.5 μL of Li-TFSI/acetonitrile (175 mg/mL) and 4 μL of tBP. Finally, a 75-nm-thick Au electrode was thermally evaporated under a vacuum of 10^−4^ Pa through a shadow mask, which defined the device area as 0.04 cm^2^. 

## 3. Results and Discussion

### 3.1. Characterization of the Perovskite Films

**Figure 2:**
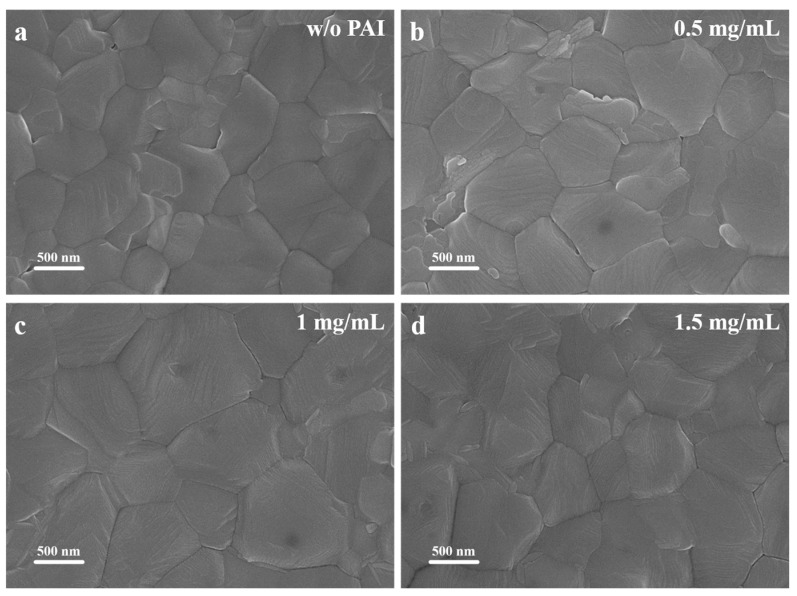
Top-view SEM (20K) images of perovskite films (**a**) without PAI treatment and with different concentrations of PAI treatment: (**b**) 0.5 mg/mL; (**c**) 1 mg/mL; (**d**) 1.5 mg/mL.

To investigate the specific effects of PAI post-treatment on perovskite films, the surface morphology was observed using scanning electron microscopy (SEM) images. Figure 2 shows the top-view SEM (20K) images of perovskite films post-treated with PAI solutions of different concentrations. As shown in Figure 2, the original perovskite film without any treatment exhibited smaller grain sizes with numerous and clear grain boundaries. After being post-treated with PAI, the grain sizes increased, reaching a maximum at a concentration of 1 mg/mL. The grain size distribution histograms calculated from the SEM images illustrate the grain size distribution for the different films, as depicted in Figure S1. The results indicate that the average grain size of the perovskite film treated with 1 mg/mL PAI reached 829 nm, while the untreated perovskite film had an average grain size of only 492 nm. The SEM results demonstrate that subsequent treatment of the perovskite film with PAI can increase the grain size and reduce the number of grain boundaries, which is crucial for enhancing the performance of perovskite solar cells.

**Figure 3:**
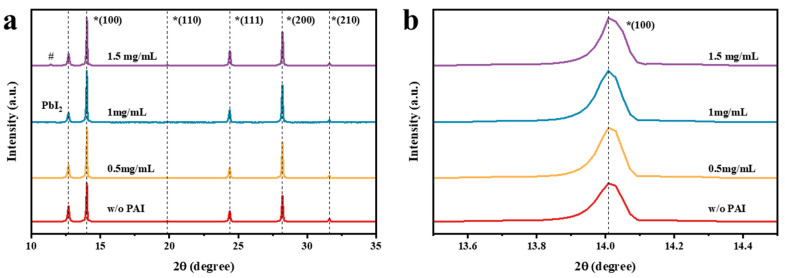
(**a**) XRD patterns of perovskite films without PAI treatment and with different concentrations of PAI treatment; (**b**) magnified XRD patterns in the (100) plane.

X-ray diffraction (XRD) was adopted to analyze the crystalline characteristics of perovskite films. The XRD patterns of perovskite films with and without PAI treatment are shown in Figure 3. It is obvious that four perovskite films exhibit typical perovskite diffraction peaks at 2θ = 14.0°, 19.8°, 24.4°, 28.2°, and 31.6°, corresponding to the (100), (110), (111), (200), and (210) planes, respectively. Additionally, all samples show residual PbI_2_ crystallinity with a diffraction peak at 12.7°. However, after treatment with PAI, the diffraction peak of PbI_2_ significantly decreased, while the intensity of the perovskite diffraction peaks increased. Here, we defined a D-index, D-index = I_PbI2_/(I_PbI2_ + I_Perovskite_), to describe the relative intensity of PbI_2_ and perovskite peaks. I_PbI2_ and I_Perovskite_ represent the diffraction intensities of PbI_2_ and the (100) plane of perovskite. The calculated results are listed in Table S1. The reduced D-index implies that PAI treatment can effectively decrease the PbI_2_ residual in the perovskite film, with the optimal effect observed at a PAI concentration of 1 mg/mL. This is mostly due to the amine groups in PAI, which can form hydrogen bonds with iodide ions and coordinate with the Pb^2+^ interstitials [31]. It has been proven that an appropriate amount of PbI_2_ residual can enhance the device performance, while an excessive amount of PbI_2_ residual will have a negative impact on the solar cell [17]. Employing PAI to passivate the perovskite film allows precise control of the PbI_2_ residual in the film, thereby improving device performance.

Moreover, it can be observed that after PAI treatment, with the increase in the intensity of the (100) plane diffraction peak, the intensity of the (111) plane diffraction peak at 2θ = 24.4° simultaneously decreased. The relative intensity changes of the diffraction peaks of different crystal planes indicate that the orientation of the perovskite thin film changed. We defined an R-index to compare the difference in film orientations: R-index = I(100)/[I(100) + I(111)], where I(100) and I(111) are the diffraction intensities of the (100) and (111) crystal planes, respectively. The calculated R-index values for each perovskite film are listed in Table S2. The R-index increased with the introduction of PAI, reaching its maximum of 83.8% when the concentration of PAI was 1 mg/mL. Conversely, the R-index of perovskite without PAI treatment was only 15.7%. These results indicate that the PAI treatment led to the preferential formation of the (100) orientation of perovskite. It has been reported that the defect density on the (100) plane of perovskite films is lower than that on other crystal planes [34]. This suggests that crystal growth oriented along the (100) plane is beneficial for reducing the defect density on the surface of perovskite films. It is noteworthy that we found that when the concentration of PAI passivation was 1.5 mg/mL, the diffraction intensity of the (100) plane decreased, while the diffraction intensity of the (111) plane increased. Additionally, a minor diffraction peak appeared at “2θ = 11.4°”, marked with “#” in Figure 3. The appearance of this diffraction peak indicates the presence of the δ-phase perovskite in the film, which implies that the excessive use of PAI can complicate the crystallization of perovskite, thereby affecting the crystallization quality of the film.

Figure 3b shows the magnified XRD image of the (100) plane; it is clearly observed that the diffraction angle of the perovskite did not shift with the use of PAI. This result supports the conclusion that the chemical composition of the film did not change, suggesting that the use of PAI likely passivates the surface defects of the perovskite film without substantially altering the point defects within the crystal. Subsequently, we calculated the full width at half maximum (FWHM) of the diffraction peaks of the (100) plane for four types of perovskite films, as shown in Figure S2. The change in FWHM can be used to assess the crystallization quality of the perovskite films. Compared with untreated films, the FWHM of passivated perovskite films significantly decreased, especially for films treated with a 1 mg/mL concentration of PAI, indicating an improvement in the crystalline quality of the perovskite.

UV–vis absorption spectra were adopted to analyze the effect of PAI post-treatment on perovskite films. As shown in Figure S3a, the absorption spectra of perovskite films passivated with different concentrations of PAI are presented. After PAI passivation, the absorption coefficient of the perovskite films slightly increased, and the absorption edge became sharper, suggesting an enhancement in the quality of the perovskite crystals with PAI passivation. Furthermore, we analyzed the absorption spectra of the films using Tauc plot curves, with the resulting spectra shown in Figure S3b. The Tauc plot analysis revealed that the optical bandgap of the perovskite remained unchanged at approximately 1.56 eV under the influence of PAI, showing no significant variation.

**Figure 4:**
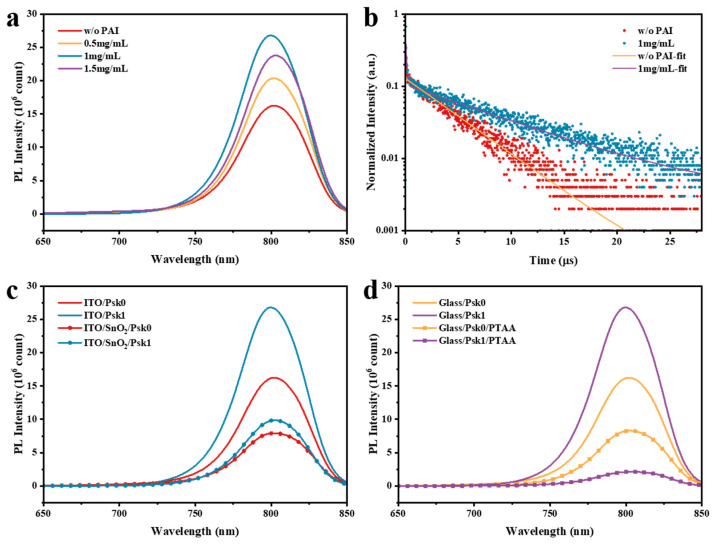
(**a**) PL of the perovskite films without PAI treatment and with different concentrations of PAI treatment deposited on glass; (**b**) TRPL of the perovskite films without PAI treatment and with 1 mg/mL PAI treatment deposited on glass; (**c**) PL of Psk0 and Psk1 perovskite films with and without SnO_2_ layer; (**d**) PL of Psk0 and Psk1 perovskite films with and without PTAA layer.

Steady-state photoluminescence (PL) spectroscopy (excited at 479 nm) and time-resolved photoluminescence (TRPL) spectroscopy (excited at 479 nm and monitored at 800 nm) were employed to further understand the properties of perovskite films. Figure 4a illustrates the PL spectra of perovskite films passivated with different concentrations of PAI deposited on glass substrates. From the PL spectra, it can be observed that the luminescence intensity of perovskite films significantly increased after PAI passivation. When the PAI concentration was 1 mg/mL, the PL intensity reached its maximum, suggesting that non-radiative recombination in the perovskite films was greatly reduced. This can be attributed to the increase in grain size, which reduces the number of grain boundaries, thereby decreasing the pathways for carrier recombination and consequently reducing the odds of non-radiative recombination in the perovskite films. Additionally, the introduction of PAI promotes the growth of perovskite crystals along the (100) orientation, reducing defects on the film surface and thus lowering non-radiative recombination. It is noteworthy that after passivating the perovskite with 1 mg/mL PAI, a blue shift in the luminescence peak occurred, as shown in Figure 4a, further indicating that surface defects of the perovskite were passivated.

Moreover, TRPL spectra of perovskite films without post-treatment and with post-treatment with 1 mg/mL PAI were tested, as shown in Figure 4b. The TRPL spectral data were fitted with a biexponential model according to Equation (1):

(1) $$I(t)=A+B_{1}exp(-t/\tau_{1})+B_{2}exp(-t/\tau_{2})$$

The average decay lifetime *τ_ave_* of the films was calculated according to Equation (2), with the relevant parameters listed in Table 1.

(2) $$\tau_{ave}=\sum B_{i}\tau_{i}^{2}/\sum B_{i}\tau_{i}$$

From the values in the table, it can be seen that after passivation with 1 mg/mL PAI, the decay lifetime of the perovskite films significantly extended from ~3.95 μs to ~8.10 μs. The increase in the decay lifetime further confirms the reduction in non-radiative recombination in the perovskite.

To investigate the impact of PAI passivation on the charge extraction capability of perovskite films, samples with charge transport materials were prepared. As shown in Figure 4c,d, we selected untreated perovskite films (labeled Psk0) and those treated with 1 mg/mL PAI (labeled Psk1), and we induced PL quenching using SnO_2_ and PTAA. The introduction of electrodes and charge transport layers makes the quenching of luminescence more related to the charge extraction of the perovskite [35,36]. From Figure 4c, it can be seen that after the introduction of SnO_2_, the PL quenching of the Psk1 sample was more pronounced. Meanwhile, as depicted in Figure 4d, the introduction of PTAA into the two perovskite samples also led to PL quenching, with Psk1 experiencing notably greater quenching compared with Psk0. This is because the photoexcited carriers in the perovskite rapidly transferred to the PTAA layer, leading to a significant decrease in PL intensity. These results indicate that PAI passivation profoundly enhances the charge extraction capability of perovskite films, especially for holes. The reason for this improvement is that PAI not only increases the grain size of the perovskite but also treats the upper surface of the perovskite films, passivating the interface defects between the active layer and the hole transport layer, thus greatly enhancing the hole extraction capability.

### 3.2. Characterization of the Perovskite Devices

**Figure 5:**
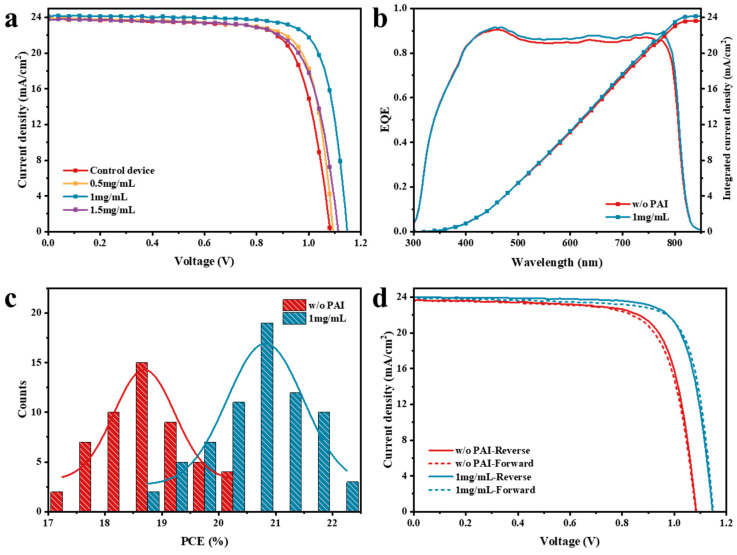
(**a**) J-V characteristic curves of devices without PAI treatment and with different concentrations of PAI treatment; (**b**) EQE spectra, (**c**) statistics distribution of PCE; and (**d**) J-V characteristic curves in reverse and forward scan directions from devices without PAI treatment and with 1 mg/mL PAI treatment.

This study further explored the impact of PAI passivation on the photovoltaic performance of perovskite solar cells. The current density-voltage (J-V) characteristics of devices were tested under standard AM 1.5 G solar simulator and 100 mW/cm^2^ light intensity irradiation, as shown in Figure 5a. The corresponding photovoltaic parameters are listed in Table 2. As can be seen in Figure 5a, compared with the untreated devices, the photovoltaic response of the PAI-passivated devices was greatly enhanced, especially in terms of open-circuit voltage and fill factor. When the PAI concentration was 1 mg/mL, the device performance was optimal, with the open-circuit voltage increasing from 1.08 V in the control group to 1.15 V, the fill factor increasing from 75.2% to 78.9%, and the power conversion efficiency improving from 19.3% to 21.9%. However, when the PAI concentration was further increased to 1.5 mg/mL, the efficiency of the device started to decline, likely due to the formation of the δ-phase perovskite. The device optimized with a PAI concentration of 1 mg/mL achieved a certified efficiency of 21%, as verified by the National Institute of Metrology (NIM), with the certification shown in the Supplement.

These results are well corroborated by the external quantum efficiency (EQE) spectra reported in Figure 5b. The EQE spectra followed a similar pattern to the absorption spectra. The integrated current density calculated from the EQE spectra showed a perfect match with respect to the one measured directly under the sun simulator. The power conversion efficiency (PCE) of approximately 50 devices without any treatment and with passivation with 1 mg/mL PAI was evaluated. The statistical distribution histogram is shown in Figure 5c. The average efficiency of the untreated control devices was about 18.5%, while the average efficiency of the devices passivated with 1 mg/mL PAI was about 21%. The results further confirm the positive effect of PAI passivation and demonstrate high reproducibility. The study analyzed the hysteresis phenomenon in perovskite solar cells by testing the control device and the 1 mg/mL device in both the forward (from 0 V to open-circuit voltage) and reverse (from open-circuit voltage to 0 V) scanning directions. The measured J-V characteristic curves are depicted in Figure 5d, and the corresponding photovoltaic parameters are listed in Table S3. The results indicate a significant reduction in device hysteresis after PAI optimization. As reported, the presence of residual PbI_2_ in perovskite is linked to hysteresis. PAI passivation effectively reduces the residual PbI_2_ content in the film to an optimal level, thus improving device efficiency and minimizing hysteresis.

**Figure 6:**
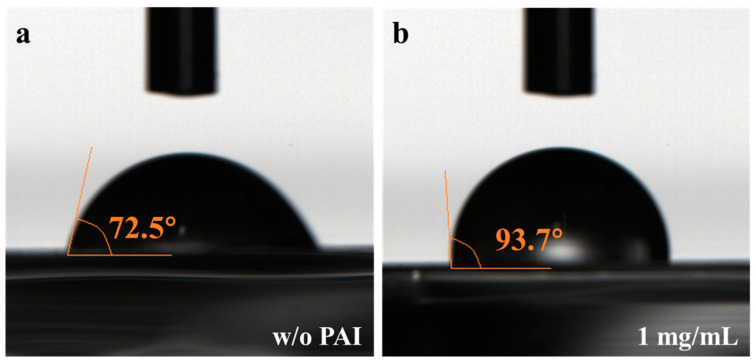
Contact angles of water on perovskite films (**a**) without PAI treatment and (**b**) with 1 mg/mL PAI treatment.

**Figure 7:**
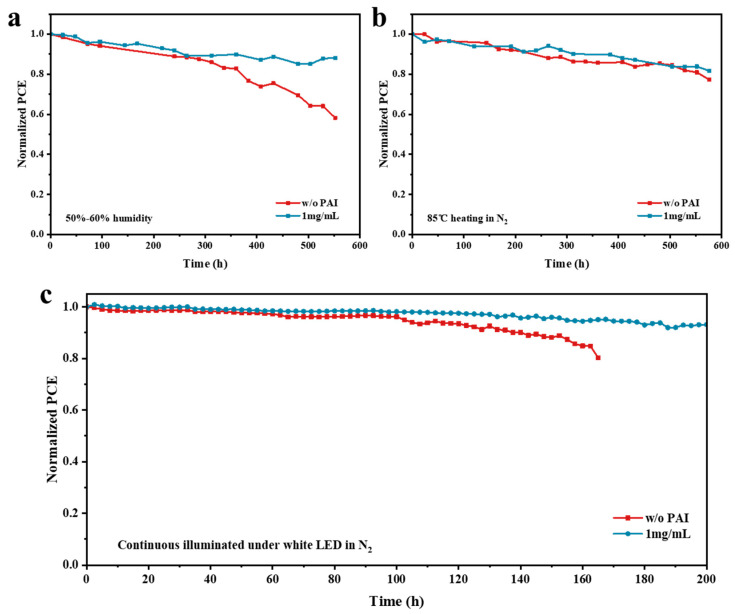
Stability test of devices without PAI treatment and with 1 mg/mL PAI treatment (**a**) stored under 50–60% relative humidity; (**b**) stored at 85 °C in N_2_; and (**c**) under continuous illumination in N_2_.

In addition to efficiency, stability is also a key factor in assessing the performance of solar cells. Perovskite materials are highly prone to decomposition under the influence of water and heat, leading to poor device stability. The contact angles between the perovskite films and deionized water were tested, with the results shown in Figure 6. Deionized water was separately dropped onto the perovskite films without passivation treatment and with passivation with 1 mg/mL PAI. After PAI treatment, the water contact angle of the film increased from “72.5°” to “93.7°”. The PA molecules remaining at the grain boundaries made the perovskite more hydrophobic, which protects the film from water erosion. Subsequently, the humidity stability of the devices was tested. Figure 7a demonstrates the stability of devices under 50–60% relative humidity. It can be observed that the stability of the devices treated with PAI was significantly enhanced, maintaining 90% of the initial efficiency after being stored for 500 h. For the device without PAI treatment, the PCE dropped to 60% of the initial value. Figure 7b shows the storage stability test of solar cells at 85 °C in N_2_. Both devices exhibited good thermal stability, with the PCE remaining at 80% of the initial value after 500 h. The study further analyzed the stability of the solar cells under continuous illumination, simultaneously testing the untreated control device and the optimal experimental devices with 1 mg/mL PAI. Both devices were placed in a nitrogen environment and continuously illuminated with white light LEDs with a power of 100 mW/cm^2^. During illumination, a bias voltage was applied to the devices at their maximum power point continuously. The J-V characteristic curves of the devices were recorded every hour. The test results are shown in Figure 7c, where a clear difference in stability between the two devices can be observed. After continuous illumination for 160 h, the efficiency of the control device degraded to 80% of the original efficiency, while the experimental device passivated with PAI maintained 90% of the original efficiency after 200 h of continuous illumination.

### 3.3. The Effect of PAI in p-i-n Device

**Figure 8:**
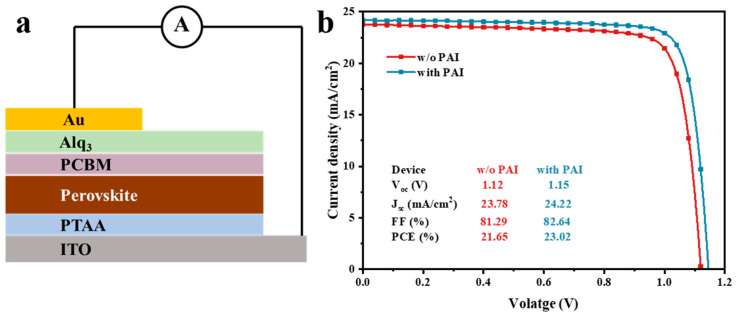
(**a**) A schematic diagram of device structure of p-i-n perovskite solar cells; (**b**) J-V characteristic curves of without and with PAI buried interface layer.

Considering the passivation effect of PAI on the perovskite film in conventional devices and the enhancement of hole transport capability, we attempted to use it in p-i-n-type perovskite solar cells to investigate the role of PAI. The experiment employed a cell structure of ITO/PTAA/Perovskite/PCBM/Alq_3_/Au, with the perovskite composition being Cs_0.05_(FA_0.92_MA_0.08_)_0.95_Pb(I_0.92_Br_0.08_)_3_, as depicted in Figure 8a. The detailed process of device fabrication is presented in the Supplement. PAI was spin-coated between the PTAA layer and the perovskite film, functioning as a buried interface layer. 

The J-V characteristic curves of the original device and the device after PAI passivation are shown in Figure 8b, with the corresponding photovoltaic parameters listed in Table S4. The devices with the addition of the PAI layer showed a certain improvement in both Voc and FF. The effect was similar to the performance enhancement observed in n-i-p-type devices. The efficiency of the optimized device increased from 21.7% in the original device to 23%. This result demonstrates the adaptability of PAI molecules. In our work, the efficiency of the p-i-n device was higher than that of the n-i-p device. This can be attributed to the use of organic charge transport materials in inverted devices, which generally possess superior charge carrier transport properties, smoother surfaces, and lower defect densities. By contrast, SnO_2_ is adopted in n-i-p devices as an electron transport layer. It is prepared by spin-coating a nanoparticle dispersion, which causes a relatively rough surface with pinholes and cracks, leading to charge carrier recombination at the interface and poor device performance [37,38,39].

## 4. Conclusions

In summary, we employed propylamine hydroiodide (PAI) for the post-treatment of perovskite films, resulting in reduced defects and suppressed recombination. Consequently, we achieved a certified efficiency of 21% in an n-i-p perovskite device. Surprisingly, PAI was also effective in p-i-n device functioning as a buried interface layer. The PCE increased from 21.65% to 23% with the introduction of the PAI layer. This method shows promise for developing low-cost, highly efficient perovskite solar cells.

## Figures and Tables

**Table 1:** TRPL lifetime parameters of the perovskite films without PAI treatment and with 1 mg/mL PAI treatment deposited on glass.

Sample	*τ*_1_ (ns)	*B*_1_ (%)	*τ*_2_ (ns)	*B*_2_ (%)	*τ_ave_* (ns)
w/o PAI	3.16	757.17	4121.74	12.99	3945.30
1 mg/mL	91.83	40.47	8382.79	12.42	8090.72

**Table 2:** Photovoltaic parameters of devices without PAI treatment and with different concentrations of PAI treatment.

Device	V_oc_ (V)	J_sc_ (mA/cm^2^)	FF (%)	PCE (%)
w/o PAI	1.08	23.79	75.18	19.32
0.5 mg/mL	1.09	23.94	77.01	20.09
1 mg/mL	1.15	24.11	78.94	21.89
1.5 mg/mL	1.11	23.76	74.37	19.62

## Data Availability

Data are contained within the article and the Supplementary Materials.

## Supplementary Materials

The following supporting information can be downloaded at: https://www.mdpi.com/article/10.3390/nano14171416/s1, Figure S1: Histograms of grain size distribution for perovskite films without PAI treatment and with different concentrations of PAI treatment; Figure S2: Full width at half maximum of the diffraction peaks of (100) planes; Figure S3: UV-vis absorption spectra of perovskite films; Table S1: D-index of perovskite films without PAI treatment and with different concentrations of PAI treatment; Table S2: R-index of perovskite films without PAI treatment and with different concentrations of PAI treatment; Table S3: Photovoltaic parameters of perovskite devices without PAI treatment and with 1 mg/mL PAI treatment at reverse and forward scan directions; Table S4: Photovoltaic parameters of p-i-n devices without and with PAI layer; Efficiency certification from National Institute of Metrology.
