# Supplementary material for: Enhancing Perovskite Solar Cell Performance through Propylamine Hydroiodide Passivation
Source: Nanomaterials (Basel). 2024 Aug 29;14(17):1416. doi: 10.3390/nano14171416 (PMC11397452; doi:10.3390/nano14171416)

# Enhancing Perovskite Solar Cell Performance through Propylamine Hydroiodide Passivation

**Fulin Sun [1], Ting Zhu [1], Chenhui Zhang [1], Yi Dong [1], Yuzhu Guo [1], Dan Li [2,*], Fangtian You [1] and Chunjun Liang [1,*]**

[1] Key Laboratory of Luminescence and Optical Information, Ministry of Education, Institute of Optoelectronic Technology, Beijing Jiaotong University, Beijing 100044, China
[2] Department of Physics, School of Physical Science and Engineering, Beijing Jiaotong University, Beijing 100044, China
* Correspondence: danli@bjtu.edu.cn (D.L.); chjliang@bjtu.edu.cn (C.L.)

## Experimental section

### p-i-n device fabrication

The pre-patterned ITO coated glass substrates were cleaned using detergent, deionized water, and ethanol sequentially and then treated by ultraviolet ozone for 20 minutes before use. For the p-i-n device, PTAA ($\overline{M_n}$<6000) was dissolved in toluene with a concentration of 2.5 mg/mL , which was then spin-coated on ITO/glass substrates at 5000 rpm for 30 s and annealed at 100 °C for 10 min. The PAI solution (2 mg/mL in DMF) was spin-coated on the PTAA layer at 5000 rpm for 30 s. The $Cs_{0.05}(FA_{0.92}MA_{0.08})_{0.95}Pb(I_{0.92}Br_{0.08})_3$ (1.2 M) precursor solution was prepared in a mixed solvent of DMF and DMSO with a volume ratio of 4:1. The perovskite films were deposited onto PAI substrates with a two-step spin coating procedure. The first step was 1000 rpm for 10 s and the second step was 4000 rpm for 20 s. 150 μL of CB was dropped on the spinning substrate during the second spin-coating step at 10 s before the end of the procedure. The substrate was then immediately transferred on a hotplate and annealed at 100 °C for 60 min. After cooling down to room temperature, the electron transport layer was subsequently deposited on top of perovskite film by spin coating at 2000 rpm for 40 s using a DCB solution which contained 20 mg/mL PCBM. Then, followed by dynamic spin-coating 1 mg/mL $Alq_3$ in ethanol at 2000 rpm for 40 s. Finally, an 80-nm-thick Au electrode was thermally evaporated under a vacuum of $10^{-4}$ Pa through a shadow mask, which defined the device area as 0.04 cm$^2$. All the procedures were completed in a $N_2$ glovebox.

**Characterization and Measurement**

The XRD patterns were taken on a Bruker D8 advance diffractometer using Cu Kα radiation (λ = 1.5405 Å). The UV–vis absorption spectra of the film specimen were obtained using a UV–vis–NIR scanning spectrometer (Shimadzu UV-3101PC). The microscopic images of the surfaces were taken via a field-emission SEM (ZEISS GeminiSEM 300) working at secondary electron mode with 20 k multiplication using an electron beam ac-celerated at 15 kV and a distant of ≈6 mm. The steady state or transient photoluminescence (PL) spectra were measured using a Horiba Nanolog FL3-2iHR fluorescence spectrometer and Horiba Deltaex ultrafast lifetime spectrofluorometer, respectively. Current-voltage (J-V) characteristics of the devices were measured using a keithley 2635B source measurement unit under an illumination of AM 1.5G solar irradiation and light intensity of 100 $mW/cm^2$ from a solar simulator (CROWNTECH SOLARBEAM-02-3A). J-V characteristics of the de-vices were measured at a scanning rate of 60 mV/s (voltage step of 20 mV and delay time of 1 s) from −1.2 to 0.1 V as reverse scan, and from 0.1 to 1.2 V as the forward scan. The EQE of the solar cells were detected with a solar cell quantum-efficiency measurement system (Zolix Solar Cell Scan 100). The contact angle was measured with an optical contact angle measuring and contour analysis systems (Dataphysics OCA 25).

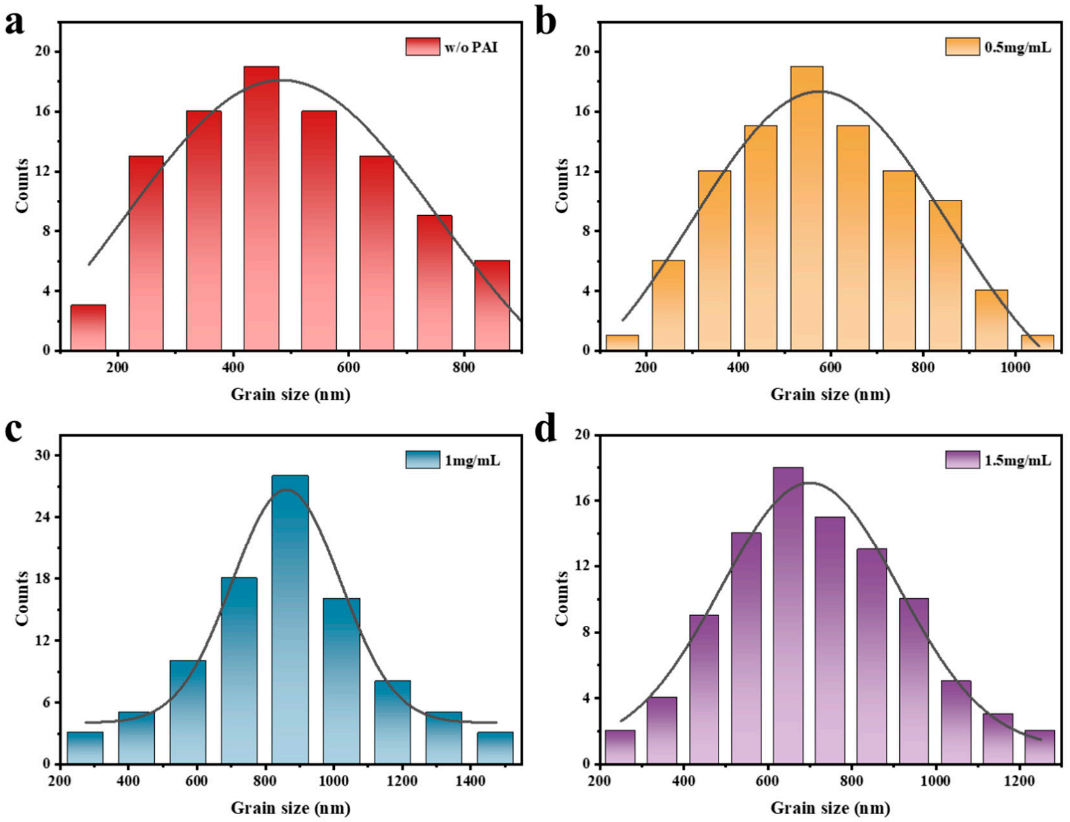


Figure S1. Histograms of grain size distribution for perovskite films without PAI treatment and with different concentrations of PAI treatment

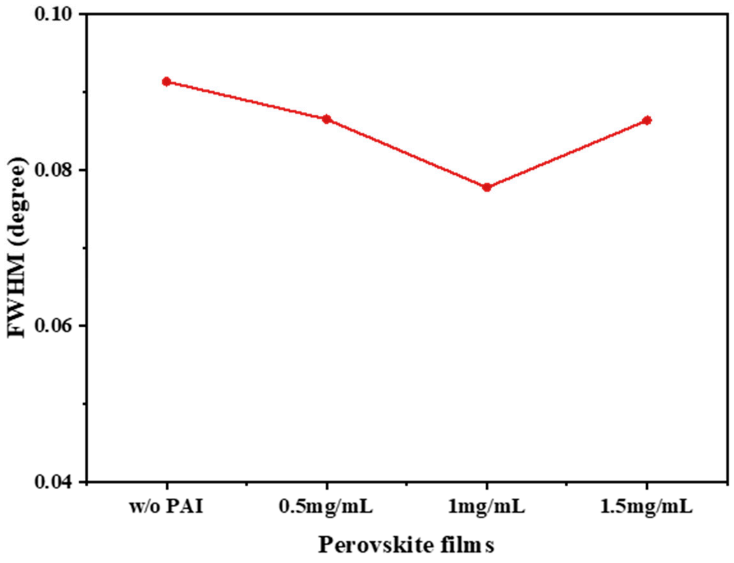


Figure S2. Full width at half maximum of the diffraction peaks of (100) planes.

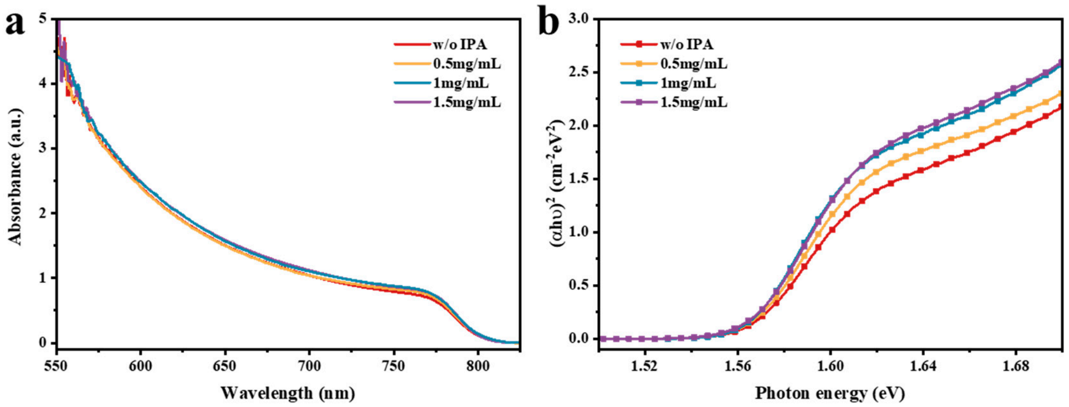


Figure S3. a. UV-vis absorption spectra of perovskite films without PAI treatment and with different concentrations of PAI treatment; b. Tauc plot curves of the absorption spectra.

Table S1. D-index of perovskite films without PAI treatment and with different concentrations of PAI treatment.

| Perovskite film | w/o PAI | 0.5mg/mL | 1mg/mL | 1.5mg/mL |
|---|---|---|---|---|
| D-index (%) | 28.6 | 20.5 | 15.9 | 19.6 |

Table S2. R-index of perovskite films without PAI treatment and with different concentrations of PAI treatment.

| Perovskite film | w/o PAI | 0.5mg/mL | 1mg/mL | 1.5mg/mL |
|---|---|---|---|---|
| R-index (%) | 75.7 | 82.9 | 83.8 | 76.2 |

Table S3. Photovoltaic parameters of perovskite devices without PAI treatment and with 1mg/mL PAI treatment at reverse and forward scan directions.

| Device | Scan directions | $V_{oc}$ (V) | $J_{sc}$ ($mA/cm^2$) | FF (%) | PCE (%) |
|---|---|---|---|---|---|
| w/o PAI | Reverse | 1.08 | 23.6 | 75.3 | 19.2 |
| | Forward | 1.08 | 23.6 | 73.4 | 18.7 |
| 1mg/mL | Reverse | 1.15 | 24.0 | 77.9 | 21.5 |
| | Forward | 1.15 | 23.8 | 78.0 | 21.4 |

Table S4. Photovoltaic parameters of p-i-n devices without and with PAI layer.

| Device | $V_{oc}$ (V) | $J_{sc}$ ($mA/cm^2$) | FF (%) | PCE (%) |
|---|---|---|---|---|
| w/o PAI | 1.12 | 23.78 | 81.29 | 21.65 |
| With PAI | 1.15 | 24.22 | 82.64 | 23.02 |

**Efficiency certification from National Institute of Metrology**

中 国 计 量 科 学 研 究 院

## Appendix: Summary of Certificate

**NIM Certificate No.: GXtc2019-1004**
**Client: Beijing Jiaotong University**
**DUT S/N：SFL20#-M2-R-02**
**Date of Test: 05/10/2019**
**Manufacturer：Beijing Jiaotong University**
**Type: Perovskite Solar Cell**
**Temperature Sensor/Control System: None**
**Mask: An aperture area of 2.167 mm$^2$ (Certificate No.: CDjc 2019-0344)**
**Environmental conditions at the time of calibration: (22.1±1) ℃, RH (41.0±2) %**

The calibration has been conducted by the PV Metrology Lab of NIM (National Institute of Metrology, China). Measurement of irradiance intensity and all other measurements are traceable to the International System of Units (SI). The performance parameters reported in this certificate apply only at the time of the test for the sample.

| Area (mm$^2$) | $I_{sc}$(mA) | $V_{oc}$(V) | $P_{max}$(mW) |
|---|---|---|---|
| 2.167 | 0.530 | 1.106 | 0.454 |
| $I_{max}$(mA) | $V_{max}$(V) | FF (%) | $\eta$ (%) |
| 0.489 | 0.930 | 77.5 | 21.0 |

**I-V Characterization Methods:**
Refer to IEC60904-1 2017: Measurement of photovoltaic current-voltage characteristics
According to JJF 1622-2017: Calibration Specification of Solar Cells：Photoelectric Properties

**Secondary Reference Cell:**
Device S/N: 81#
Device Material: Mono-Si

**Solar Simulator:**
Classification: AAA (Double-light source: Xeon and Halogen)
Total irradiance: 1000 W/m$^2$ based on $I_{sc}$ of the above Secondary Reference Cell.

| Issue Date | 05/10/2019 |
|---|---|

中 国 计 量 科 学 研 究 院

**NIM Certificate No.: GXtc2019-1004**

**DUT S/N：** SFL20#-M2-R-02

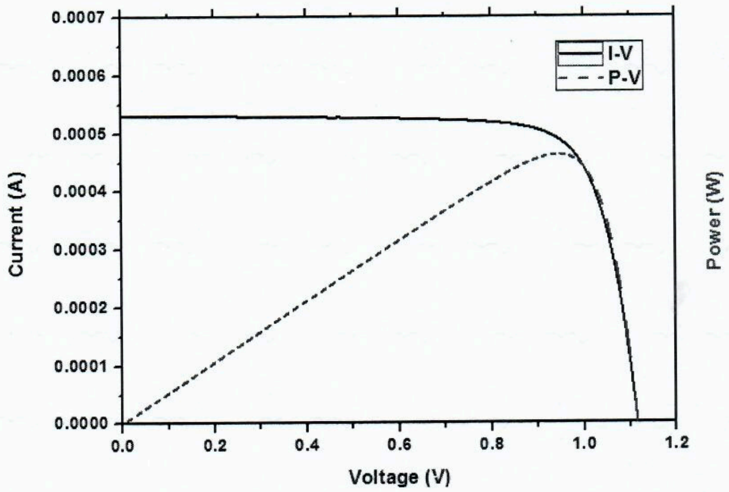

Supplement: Supplementary file 1 [file nanomaterials-14-01416-s001.zip › nanomaterials-3081091-supplementary.pdf]
